# Factors affecting consumer acceptance of electronic cash in China: an empirical study

**DOI:** 10.1186/s40854-021-00312-7

**Published:** 2022-02-01

**Authors:** Bo Qu, Li Wei, Yujia Zhang

**Affiliations:** 1 Statistics Department, National Internet Finance Association of China, Beijing, 100032 China; 2 Research Institution, The People’s Bank of China, Beijing, 100032 China; 3 School of Finance, Renmin University of China, No. 59 Zhongguancun Street, Haidian Dist., Beijing, 100872 China; 4 China Financial Policy Research Center, Renmin University of China, No. 59 Zhongguancun Street, Haidian Dist., Beijing, 100872 China; 5 China Insurance Institute, Renmin University of China, No. 59 Zhongguancun Street, Haidian Dist., Beijing, 100872 China

**Keywords:** Electronic cash acceptance, UTAUT, SEM, DCEP

## Abstract

This paper proposes and validates a comprehensive model of consumer acceptance in the context of offline e-cash payment. It modifies the unified theory of acceptance and the use of technology model (UTAUT) with constructs of perceived security, cost of use, and government policy. Data collected from 4428 questionnaires about users’ attitudes toward e-cash is used to apply a structural equation model which, in turn, assesses the predictive model. The empirical results indicate that perceived security and cost of use are beneficial extensions to the traditional UTAUT model, and intention is a key antecedent to users’ actual utilization of e-cash. In addition, the demographic moderators are found to have significant effects on the relations among the variables. These results are useful to e-cash development and significant to the issue of Digital Currency Electronic Payment.

## Introduction

Technology development has resulted in the emergence of cashless societies in countries across the world. Electronic cash (e-cash), also known as digital cash, was proposed as one of the most important forms of electronic payment. There are several different types of e-cash in the world. The type studied in this paper refers specifically to card-based cash, which is a small payment function based on the application of a financial IC card.^1^ Without having to enter a password or signature, a cardholder can use it for immediate purchase, just contacting the offline sales terminal (Li 2014). For security consideration, cardholders are just allowed to make a deposit in the e-cash account within a limit amount. E-cash is a technology innovation with enormous potential to improve the payment experience of consumers and streamline the governments’ public services.

E-cash is different from other e-payments and is used to meet an increasing demand for quick micropayment. It facilitates safe, quick, and anonymous offline payments for customers. Based on intelligent chips and offline technology, e-cash addresses the problems of unavailability of internet connection and slow speed of online transactions, which are issues typically associated with some traditional bank cards, online payment, and mobile payment. It also enables the cardholders to pay without signatures and other authentications and does not entail the disadvantages of prepaid cards, which are typically unavailable for trans-regional and trans-industry use. Furthermore, e-cash stored on integrated circuit cards notably reduces cash-handling fees, replaces change and coins, and avoids counterfeit problems with asymmetric encryption and other advanced security technology standards.

Despite the numerous e-cash advantages and the widespread enthusiasm, its usage is still relatively low. This paper contributes to the few previous academic studies that address the issues around increasing the adoption of e-cash; however, it differs from that literature by assessing what determines the consumer’s intention to use e-cash. Those studies concentrate on improving the technical aspects of e-cash including protocol engineering, information security, cryptography, and system engineering (Saputra et al. 2015). Besides studying technical aspects, it is significant to analyze the consumer’s acceptance of e-cash. Inadequate user acceptance seems to be the biggest obstacle here as some individuals still do not want to switch to e-cash due to their payment habits or perceived security considerations (Sigar 2016).

This study is different from other e-cash adoption studies because it extends the traditional UTAUT model with constructs of perceived security, government policy, and cost of use, to examine the motivations and intensions of e-cash. Perceived security and cost of use are examined to be key factors in many researchers’ UTAUT model to study the adoption of other e-payments (Khalilzadeh et al. 2017; Karrar et al. 2019). As e-cash is a typical kind of e-payment, these two factors are considered to be significant in the proposed model. Meanwhile, similar to Digital Currency Electronic Payment (DCEP), e-cash in this paper has not yet changed the way central banks issue credit currency (Li 2018). This issue makes its usage more susceptible to government policy than the e-payments studied in previous research (Shin 2009; Thusi and Maduku 2020). Those payments are managed by a third party, such as a mobile wallet. Therefore, it is important to integrate these factors into the UTAUT model and investigate users’ concerns over e-cash.

Moreover, the volume of the exclusive data contained in this paper is desirable to analyze consumer acceptance of e-cash. This paper conducts an empirical assessment of the predictive model using a structural equation model (SEM). The sample size is proved suitable to SEM and far exceeds the size in other studies (Wei et al. 2021; Arfi et al. 2021). This paper also studies the effects of demographic moderators on the relations among the variables.

E-cash is an early exploration in the process of changing from hard cash to digital cash (Li 2014). The findings in this paper can be helpful to e-cash application and can provide valuable experience for the future of DCEP. Although DCEP is actively pilot testing in China now, it will take a long time to see the results and collect research data. Based on the e-cash data, helpful suggestions could be obtained.

The structure of this paper is as follows: Sect. 2 provides the literature review of the model used to investigate the e-cash adoption. Section 3 presents the hypotheses of the model to be tested. Section 4 performs the research design, which includes survey methodology, the statistical analysis, and testing of the acquired data. Section 5 gives the results of the empirical test together with the influence of demographic characteristics on e-cash use. Finally, Sect. 6 presents the conclusions of this paper, the limitations, and future research suggestions.

## Theoretical background

### The e-cash payment in China

E-cash has been introduced in many countries like China (Li 2014), Indonesia (Satupra and Supangkat 2014), Japan (Nishi et al. 2015), and London (Fujiyama and Cao 2016). This is especially true in China, a country that is at the forefront of developing digital currencies in the world. Since 2013, cities such as Ningbo, Chengdu, and Changsha, have been selected by the Chinese Central Bank as pilot cities for the application of e-cash use. Hundreds of millions of IC cards have been issued with e-cash functions. E-cash is used in 28 major industries, covering public transport, cultural education, medical and health care, social security, public welfare, and other fields. During the first quarter of 2018, the public transport transaction amount in Guizhou achieved 65.33 million yuan. E-cash has achieved good social and economic benefits and provides an ideal venue for the study of consumer acceptance of e-cash.

E-cash is an early exploration of digital cash popularity in China (Li 2014). It offers greater convenience, notwithstanding the rise of mobile payments. Like DCEP, bank card-based e-cash is a kind of credit currency issued by central banks. Similar to the paper currency circulation, the framework of e-cash is from central banks to commercial banks to the public” (Li 2018), which makes it easier for the central bank to supervise. On the contrary, internet or mobile payments representing the specific issuers’ credit and decentralized virtual coins (such as bitcoins) will probably affect the current currency supply system, which is comprised of central banks–commercial banks, and disrupts traditional currency circulation (Li 2018). The Central Bank has to seriously consider its impact on the operation of the payment system, the monetary system, and the stability of the financial system, and to more actively put forward countermeasures to optimize and upgrade the legal tender issuance and circulation system. Therefore, these findings of e-cash adoption should be of interest to both academics and practitioners.

### Technology acceptance and use theories

The continuous advancement and popularization of information technology has injected new vitality into society, but in many cases the introduction of new technologies has not achieved the expected results. This problem has drawn the attention of many scholars and they have put forward different explanations.

From the user's point of view, the theory of technology acceptance investigates how beliefs and attitudes determine a consumer’s intention to use new technology. The Theory of Reasoned Action (TRA), the Theory of Planned Behavior (TPB), and Innovation Diffusion Theory (IDT) are credited with being the first theories to explain technology adoption and acceptance (Arfi et al. 2021). Ajzen and Fishbein (1977) proposed TRA, which takes attitudes and subjective norms as determinants of behavior. Ajzen (1991) then extended the TRA by adding the variable of Perceived Behavior Control in TPB. The IDT proposes innovative features (Rogers 2003).

Based on TRA, the Technology Acceptance Model (TAM) was developed to explain and predict user adoption in various information systems (Nur and Panggabean 2021). It was an early attempt to apply psychological factors to information systems and computer adoption (Shin 2009). The TAM assumed that perceived usefulness and ease of use are primary factors in an individual’s attitude toward using technology (Davis 1989). Compared with TRA or TPB, TAM can better explain people's intention to use new technologies (Leong et al. 2013). Previous studies have demonstrated the validity of the framework in explaining technology acceptance. However, in recent years, TAM has been criticized for its simplicity (Shachak et al. 2019), and researchers tried to extend TAM with incorporated additional variables for specific contexts (Zhao and Bacao 2021).

As a result, the Unified Theory of Acceptance and Use of Technology (UTAUT) has been proposed as an extension of TAM. Proposed by Venkatesh et al. (2003), the UTAUT model integrates TRA, TPB, TAM, motivation models, and social cognitive theories into the framework. The aim is to explain the user's intention to use a new system and to explain the subsequent use behavior (Nur and Panggabean 2021). The model indicates that four core factors influence user acceptance: Performance Expectancy, Effort Expectancy, Social Influence, and Facilitating Conditions (Zhao and Bacao 2021; Venkatesh et al. 2003). In addition, the importance of control variables was tested by researchers with the UTAUT model.

### UTAUT framework in e-cash payment

In order to study the adoption of e-cash, the UTAUT is a more suitable model. It has the following advantages over other theories. First, the UTAUT model was proposed by integrating several developed theories, and its prediction efficiency is higher. The framework includes both host and control variables, and its prediction efficiency is 70% higher than that of the TAM model (Christine 2018). The model tested in healthcare settings explained 70% of the variance in Behavioral Intention (BI) and about 50% in actual use (Cimperman et al. 2016; Duarte and Pinho 2019).

The UTAUT model recognizes the importance of including a social influence component in the model, which is neglected by the TAM model (Nur and Panggabean 2021). Previous studies verified the importance of social influence in technology adoption, such as mobile learning (Slade et al. 2015) and mobile payment (Ai-Okaily et al. 2020). As a typical payment method, e-cash is frequently used by people in their daily lives to make payments. Social influence might be a key factor in e-cash adoption. It is more suitable to use the UTAUT model, including social influence to study the issue.

Based on the character of e-cash, this study argues that the traditional UTAUT model may have a limited ability to explain e-cash adoption. The model has been extended successfully in different contexts to study the adoption of many technologies and information systems (Al-Qaysi et al. 2020). In several research projects, the perceived security is found to be a key factor that impacts the consumer’s acceptance of a new payment technology (Khalilzadeh et al. 2017; Johnson et al. 2018; Shao et al. 2018), especially in financial issues. Therefore, this study tries to validate the perceived security as a key factor in e-cash acceptance. Moreover, the TAM assumes that no barriers can stop an individual from using a technology if he or she has chosen to do so (Shin 2009). However, the cost of use is normally a key factor that prevents people from using a technology. In order to better explain e-cash adoption, this paper further extended the traditional UTAUT model with cost.

## Hypotheses

**Fig. 1:**
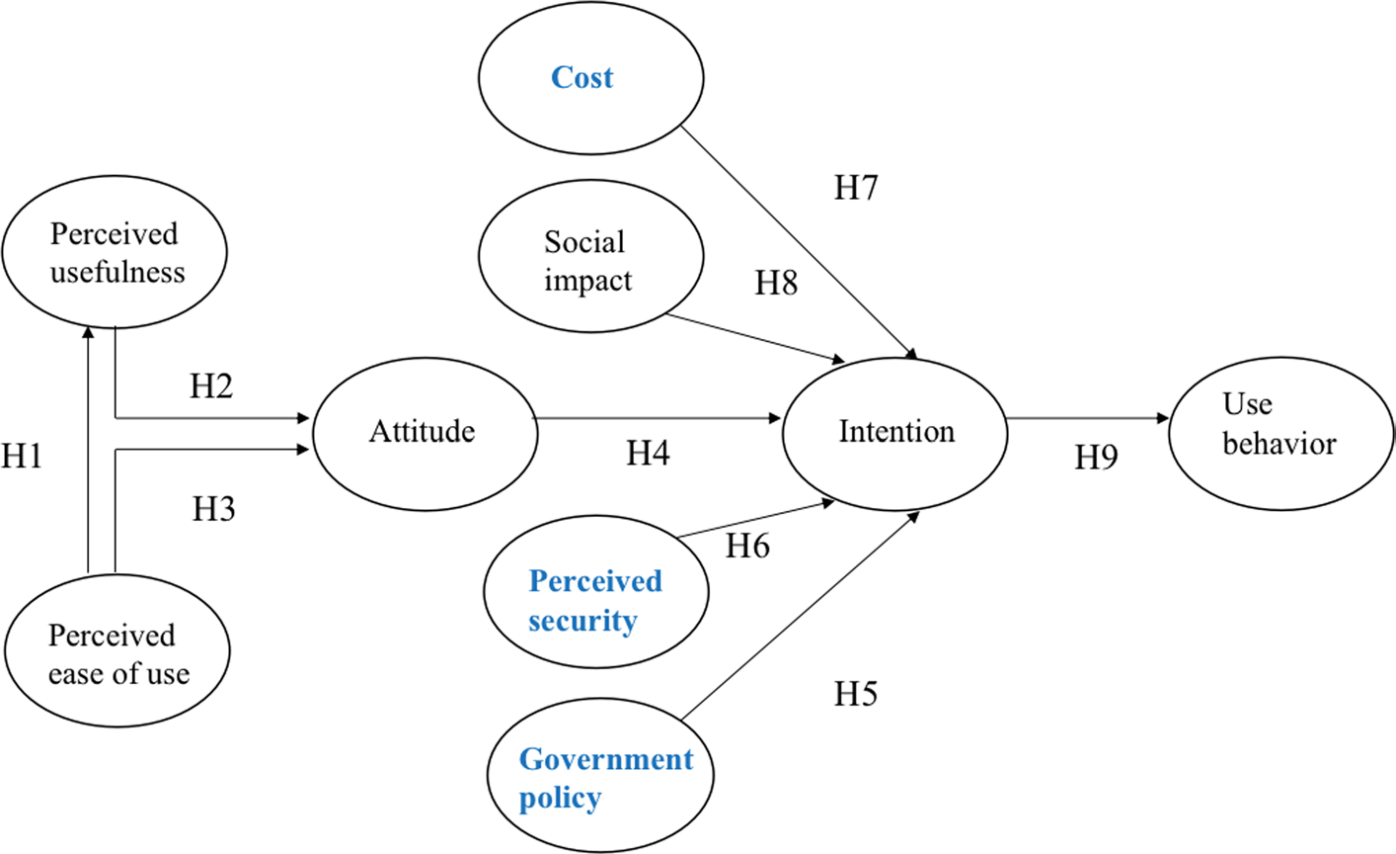
Proposed research model

This study modifies the original UTAUT model proposed by Venkatesh et al. (2003) with constructs of perceived security, cost of use, and government policy. The model is shown in Fig. 1, including e-cash perceived ease of use, perceived usefulness, attitude toward e-cash, e-cash usage intentions, and actual use behavior. The variables gender, age, income, and education are posited to moderate the impact of the key constructs. The extended indicators are changed into bold type.

### Perceived ease of use and perceived usefulness of e-cash

The perceived usefulness of e-cash (PU) refers to the degree to which users can believe in the use of e-cash services to improve the consumer experience (Venkatesh et al. 2003). The perceived ease of use (PEOU) of e-cash refers to the extent to which users do not need to spend extra effort on using e-cash (Davis 1989). Because the main application scenario of e-cash use is the general public’s rapid small payments (Li 2014), the ease of use of it will seriously restrict its usefulness.

#### **Hypothesis 1 (H1)**

The PEOU of e-cash has a significant positive impact on the PU of e-cash.

### Perceived ease of use, perceived usefulness, and attitude

The attitude toward e-cash is defined as the extent to which a person has an assessment of behavior, such as “like” (positive emotional tendencies) and “dislike” (negative emotional tendencies). The PEOU and PU of e-cash would increase the user's preference for it, which is certainly a positive emotional tendency. E-cash can significantly improve the user's consumption experience, such as solving the inconvenience of cash payments, change, storage, and other inconveniences, as well as the payment of counterfeit currency, damaged banknotes, and dirty banknotes. Besides, based on advanced smart card technology, e-cash can realize fast billing and payment without networking and can be directly connected to the contactless terminal to realize the transaction, namely, “Flash Pay” (Li 2014).

#### **Hypothesis 2 (H2)**

The PU of e-cash significantly positively affects users' attitudes toward e-cash.

#### **Hypothesis 3 (H3)**

The PEOU of e-cash significantly positively affects users' attitudes toward e-cash.

### Attitude and intention

According to the UTAUT model, attitudes toward behavior refer to positive or negative feelings when a person engages in the behavior. From the perspective of consumer psychology, only consumers who like e-cash, can have enthusiasm for its use, which means that the attitude toward e-cash significantly positively affects the intention of using e-cash. Based on the extensive use of the UTAUT model in new technology areas, it can be expected that the causal relationship found in UTAUT is equally applicable to e-cash. And the relationship between attitude and intention has been proven (Shin and Kim 2008). A lot of literature support a significant positive relationship between attitudes toward mobile payment technologies and the use of mobile payment technologies (Shin 2009).

#### **Hypothesis 4 (H4)**

Attitude toward e-cash has a significant positive impact on the intention to use e-cash.

### Perceived government policy

Perceived government policy means that consumers feel that the government provides adequate policy support for the implementation of e-cash. Government policies usually imply future developing trends, or areas that will be supported, such as tax incentives and subsidies. It may also affect people's acceptance of new technologies to some extent.

In China, the government has strong credibility and the ability to allocate resources. Therefore, individuals can confidently feel supported through the policy of government, which makes them believe that e-cash is beneficial to users in the long run and that the technology will have wider applications in the future. Thus, they will be more willing to use e-cash. Developed by the People’s Bank of China, e-cash is more susceptible to government policy. Therefore, it is important to integrate the perceived government policy into the UTAUT model and to investigate users’ perceived government policy concerns over e-cash.

#### **Hypothesis 5 (H5)**

Perceived government policy significantly positively affects the intention to use e-cash.

### Perceived security of e-cash

The perceived security of e-cash means that users believe that it is safe to use e-cash. Many researchers have proved that the perceived security of e-cash is important in the adoption of electronic payments. Shin (2009) indicated the importance of perceived security and trust for mobile wallets, and Khalilzadeh et al. (2017) demonstrated strong evidence of the effects of security and trust on customers' intentions to use mobile payment technology. In terms of e-cash, the perceived security is primarily based on consumer perceptions of its reliability and privacy. There is suspicion of the stability of mobile networks and e-cash transaction systems, and many consumers are concerned about the security of e-cash.

#### **Hypothesis 6 (H6)**

The perceived security of e-cash significantly positively affects the intention to use e-cash.

### Perceived cost

The perceived cost of using e-cash is the total amount of money that needs to be spent to use it. In the previous research, the perceived costs were regarded as frequent factors that were used to extend the UTAUT model to examine the M-payment adoption (Karrar et al. 2019). It is understandable that only when the cost of using e-cash falls to an acceptable level, will the user consider adopting the service.

#### **Hypothesis 7 (H7)**

E-cash usage costs significantly affect the use of e-cash.

### Social impact

Social impact refers to the attitude toward user behavior from those who are very important to them (such as parents, friends, or leaders in life). This definition is derived from the general definition of social impact in the field of mobile services by Nysveen et al. (2005). Social impact can speed up the individual’s acceptance of new technologies. Nysveen et al. (2005) also pointed out that when using a mobile service in a public environment, people first observe the behavior of others and are influenced by others. It is demonstrated by Verkasalo et al. (2010) that social impact has a significant positive effect on smart-phone acceptance. Wei et al. (2021) also shows that social influence has a positive influence on the younger generations’ BI to adopt mobile payment.

#### **Hypothesis 8 (H8)**

Social impact significantly positively affects the intention to use e-cash.

### Intention

Intention is the core concept in the UTAUT model (Venkatesh et al. 2003). It is the user's willingness to use e-cash, such as a strong desire to use it or the hope to be able to use it occasionally. The willingness to use new technologies will largely motivate the actual use of the technology. Jaradat et al. (2013) used e-commerce acceptance as the main object and found that the use of e-commerce intentions determines the actual use of e-commerce. Based on UTAUT, TTF, and other models, verified that the intention to use mobile banking directly affects the actual use of mobile banking.

#### **Hypothesis 9 (H9)**

The intention to use has a significantly positive effect on the actual use of e-cash.

## Research design

This study measures the acceptance of e-cash by choosing the consumers in the pilot cities of e-cash as the survey objects. In 2013, cities such as Shanghai, Ningbo, Chengdu, Changsha, and Guiyang were been selected by the Chinese Central Bank as the pilot cities for the introduction of e-cash. This section shows the survey methodology measurements and data analysis.

### Survey methodology

The survey design method consists of three phases and largely follows the method used by Shin (2009). First, the research model was augmented with individual in-depth interviews. In the interviews, undergraduate students and possible customers of commercial banks in the pilot cities were asked to explain their opinions of e-cash, how they currently use e-cash, and what factors would influence their use of e-cash in the future. The goal of the individual interviews was to test and augment the research model to identify items missing from the UTAUT. Second, the survey questionnaire was developed through several rounds of deliberation by an expert panel consisting of university researchers and e-cash systems experts. Finally, a pre-test was conducted prior to the use of the survey. The questionnaire was tested among possible users to reduce possible ambiguous questions and the wording was modified, based on the pilot test outcomes.

Ultimately, there are three sections in the questionnaire. After a welcome note explaining that the respondents’ answers are important for public policy, our survey was divided into three parts. The first section explains the key words in the survey, such as PEOU, PU of e-cash, attitude, intention, perceived government policy, perceived security of e-cash, cost, social impact, and so on. The second section is aim to collect the demographic information of the respondents, such as age, gender, education, income and so on. The last section contained the core information of the survey. The finished questionnaires were printed and handed out to the respondents in the e-cash pilot cities in China.

The sampling strategy was a non-probability convenience sample collected in cooperation with the bank outlets of several commercial banks in the pilot cities. The respondents were applicants for financial IC cards. By the time the survey ended, 5209 responses were returned and of these 4428 qualified for statistical analysis. The response rate was 80%, an acceptable rate in survey methodology. In order to use the maximum likelihood estimation method, Hair et al. (2010) suggested that the minimum sample size is 100. Hence, the sample size contained in this paper is desirable for the statistical analysis and provides a database for the use of structural equations (SEM) to analyze consumer acceptance of e-cash.

Table 1 shows the sample description. It indicates that the respondents are almost evenly distributed in the pilot cities, which ensured the objectivity of the survey results. Among the respondents, nearly 70% were between the ages of 20 and 40 and more than 60% respondents had a bachelor’s degree or above, even more than 50% of respondents earned between 30,000 and 80,000 yuan. This is understandable because many new technology users are young and educated. Also, the demographics may have resulted from pre-screening procedures that selected users of substantial experience with e-cash usage. Thus, the chosen sample reflects the general population for e-cash services.

**Table 1:** Sample description (total = 4428)

Variables	Categories	Frequency per category	%
A. City	Shanghai	912	20.6
	Changsha	884	20
	Ningbo	863	19.5
	Guiyang	873	19.7
	Chengdu	896	20.2
B. Age	Under 20 (age = 1)	77	1.7
	20–30 (age = 2)	1663	37.6
	30–40 (age = 3)	1352	30.5
	40–50 (age = 4)	936	21.1
	Over 50 (age = 5)	400	9.0
C. Gender	Male (gender = 1)	2256	50.9
	Female (gender = 2)	2172	60.5
D. Income (k)	Less than 30 (income = 1)	656	14.8
	30–50 (income = 2)	1546	34.9
	50–80 (income = 3)	768	17.3
	80–100 (income = 4)	491	11.1
	More than 100 (income = 5)	967	21.8
E. Education	High school or –(education = 1)	1589	35.9
	Undergraduate (education = 2)	2681	60.5
	Postgraduate (education = 3)	158	3.6

### Measurements

To secure internally consistent measures, appropriate items were selected in the investigation. Different items were matched with different behaviors, and all the survey questions were multiple choices. In the final questionnaire, the different items assessing a given construct were separated and presented in nonsystematic order. They were interspersed with items for other constructs. In our interviews, the interviewees are asked to answer survey questions based on the direct measures from Ajzen (2002).

All indicators are mainly based on the existing research results. The measurement items were developed from previously validated measures and were carefully restated to reflect the characteristics of e-cash. The items used a 7-point Likert-type scale (ranging from “strongly disagree” to “strongly agree”) drawn from previously validated instruments, as shown in Table 2.

**Table 2:** Measurement items

Indicators	Measurement items	References
Perceived ease of use (PEoU)	[PEoU1] I find e-cash to be easy to use [PEoU2] Interacting with E-cash does not require a lot of my mental effort [PEoU3] Learning to use E-cash would be easy for me [PEoU4] I find it easy to use E-cash [PEoU5] It would be easy for me to become skillful at using E-cash	Davis (1989) Venkatesh et al. (2003)
Perceived security (PS)	[PS1] I feel secure using E-Cash [PS2] I believe that using E-cash is secure [PS3] I think that it is secure to use E-cash	Yenisey et al. (2005)
Attitude to e-cash (A)	[A1] I think that using E-cash is a good idea [A2] In my opinion, using E-cash is beneficial to me [A3] I have positive perception about using E-cash [A4] I believe that using E-cash is a good idea [A5] I feel that using E-cash is beneficial to me	Shin and Kim (2008) Shin (2009)
Social influence (SI)	[SI1] People who are important to me think that I should use E-cash [SI2] People who are familiar with me think that I should use E-cash [SI3] People who influence my behavior think that I should use E-cash [SI4] Most people surrounding with me use E-cash	Foon and Fah (2011) Venkatesh et al. (2003)
Cost of use (Cost)	[Cost1] I think using E-cash is costly [Cost2] I believe using E-cash costs a lot [Cost3] In my opinion, It is expensive to use E-cash	Karrar et al. (2019)
Perceived government policy (GP)	[GP1] I think the government policy encourage me to use e-cash [GP2] I believe using E-cash is encouraged by the government policy [GP3] In my opinion, the government policy is beneficial to using E-cash	Government has strong credibility and ability to allocate resources. Feeling support from government policy could bring confidence to individuals
Perceived usefulness (PU)	[PU1] I believe E-cash to be useful in my life [PU2] I think E-cash to is beneficial to me [PU3] I find e-cash to be useful in my life	Davis (1989), Venkatesh et al. (2003)
E-cash usage Intentions (Intention)	[Intention1] Given the opportunity, I will use E-Cash [Intention2] I am willing to continuously use E-Cash [Intention3] I am open to using E-Cash [Intention4] I intend to continuously use E-Cash	

### Data analysis

For analysis of descriptive statistics, SPSS 19.0 was used. Amos 19.0 is used for factor analysis, reliability analysis, and the SEM. To meet the requirements of the SEM, this section carried out a comprehensive test on the data set used, including a differential validity test, a reliability test, a convergence validity test, a content reliability test, and a fit degree test.

All the variables used in this paper are mainly based on the existing research results (Table 2), so they meet the criteria of content validity. The ${ }\chi^{2} - difference$ statistic proposed by Bagozzi and Phillips (1982) was used to examine the discriminant validity of latent variables. The test algorithm involved two sets of analysis, a free model and a restricted model. In the free model, the correlation between all potential variables was calculated based on the actual situation of the data. In each of the restriction models, the correlation coefficient between two potential variables was specified as 1. If the values of $\chi^{2}$ of the free model and the restricted model have statistically significant differences, then the two potential variables specified have sufficient discriminant validity. The *p* value of all $\chi^{2} - difference$ tests $\chi^{2} - difference$ is less than 0.001, so all variables have sufficient discriminant latent validity (Table 3).

**Table 3:** Discriminant validity

	PEOU	Attitude	PS	SI	Cost	PU	GP
Attitude	19.53						
PS	134.21	100.28					
SI	199.09	148.71	216.21				
Cost	771.78	697.19	518.03	636.33			
PU	98.40	85.88	88.22	226.26	585.42		
GP	132.22	117.42	238.22	242.69	765.00	109.84	
Intention	111.81	52.68	142.51	299.42	768.52	117.81	268.02

All the *p* values of ${\upchi }^{2} - difference$ test are less than 0.001

Alpha (${\upalpha }$) (Cronbach, 1971) and combined reliability (CR) (Nunnally, 1978) were adopted to test the reliability of the data. Table 4 shows that the value of α and the CR of each latent variable have appropriate reliabilities, both values were greater than 0.7. To exam the convergence validity of latent variables, Fornell and Larcker (1981) pointed out that three conditions should be satisfied. First, the model’s factor loading should be more than 0.5 and the ideal value is above 0.7. Second, the combined reliability (CR) should be greater than 0.7. Third, the average variance extraction should be greater than 0.5. It is shown in Table 4 that the latent variables used in this paper are in full compliance with the convergence validity criteria.

**Table 4:** The reliability and validity of the measurement instrument

Latent variables	Observable variables	Factor loading	α	CR	AVE
Perceived ease of use (PEoU)	PEoU1	0.88	0.96	0.99	0.81
	PEoU 2	0.89			
	PEoU 3	0.92			
	PEoU 4	0.91			
	PEoU 5	0.91			
Perceived security (PS)	PS1	0.88	0.92	0.99	0.80
	PS2	0.90			
	PS3	0.90			
Attitude to e-cash (A)	A1	0.85	0.95	0.99	0.77
	A2	0.88			
	A3	0.90			
	A4	0.90			
	A5	0.91			
Social influence (SI)	SI1	0.89	0.95	0.99	0.81
	SI2	0.90			
	SI3	0.91			
	SI4	0.91			
Cost of use (Cost)	Cost1	0.80	0.88	0.97	0.72
	Cost2	0.88			
	Cost3	0.86			
Perceived government policy (GP)	GP1	0.88	0.92	0.98	0.78
	GP2	0.89			
	GP3	0.88			
Perceived usefulness (PU)	PU1	0.87	0.92	0.99	0.8
	PU2	0.91			
	PU3	0.91			
E-cash usage intentions (Intention)	I1	0.86	0.94	0.98	0.76
	I2	0.89			
	I3	0.82			
	I4	0.91			
	I5	0.87			

The model is effective when the model has sufficient explanatory power for the dataset, i.e., the model has a model-sufficient fit. Table 5 shows that all the fitness indexes in the consumer acceptance model for e-cash established in this paper is larger than the recommended value of the relevant literature. So, the established model is suitable for the dataset used in this paper.

**Table 5:** Fit indices for the measurement model and structural model

Fit statistic	Recommended value and resource	Model	Support
GFI	> 0.90 (Bagozzi & Yi, 1988)	0.92	Yes
AGFI	> 0.80 (Etezadi-Amoli & Farhoomand, 1996)	0.91	Yes
NFI	> 0.90 (Hu & Bentler, 1999)	0.92	Yes
CFI	> 0.92 (Hair et al., 2010)	0.97	Yes
IFI	> 0.90 (Bentler, 1989)	0.97	Yes
TLI	> 0.90 (Hair et al., 2010)	0.96	Yes
PGFI	> 0.50 (Bentler, 1994)	0.76	Yes
PCFI	> 0.50 (Bentler, 1994)	0.85	Yes
PNFI	> 0.50 (Bentler, 1994)	0.84	Yes
SRMR	< 0.08 (Hair et al., 2010)	0.02	Yes
RMSEA	< 0.05–0.08 (Herry & Stone, 1994; Byrne, 2001)	0.05	Yes

*GFI* goodness-of-fit index, *AGFI* adjusted goodness-of-fit index, *NFI* normed fit index, *CFI* comparative fit index, *IFI* incremental fit index, *RFI* relative fit index, *PGFI* parsimony goodness-of-fit index, *PCFI* parsimonious comparative fit index, *PNFI* parsimonious normed fit index, *SRMR* standard root mean square residual, *RMSEA* root mean square error o

## Empirical results

**Fig. 2:**
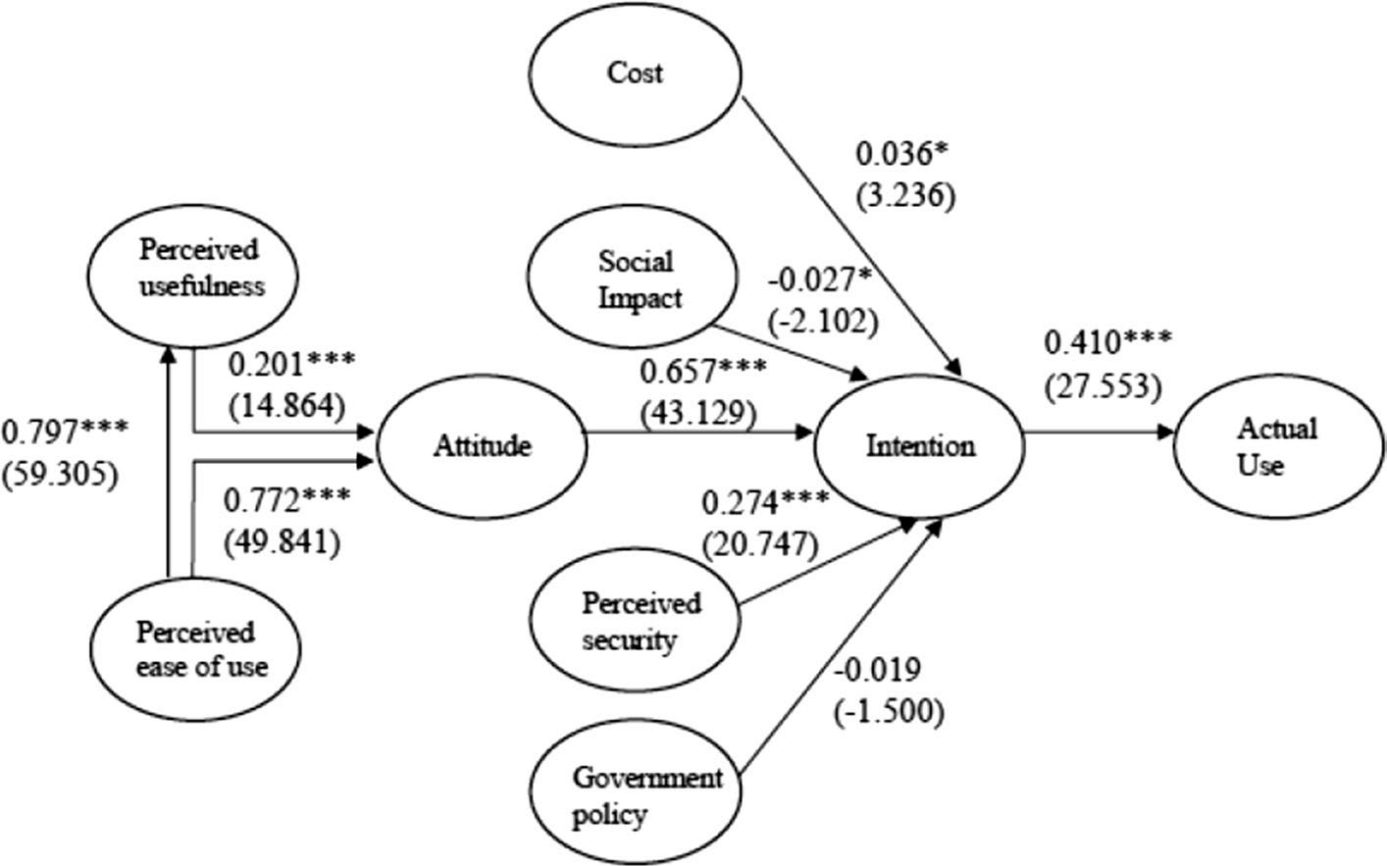
Result of the research model

As shown in Table 6 and Fig. 2, the paper examines the structural relationships of the proposed model by calculating the path coefficients between the latent variables.

**Table 6:** Summary of the hypothesis test

Hypothesis	Coefficient	S.E	C.R	*p* value	Support
${\text{H}}_{1} :{\text{PEoU}} \to {\text{PU}}$	0.797***	0.013	59.305	< 0.001	Yes
${\text{H}}_{2} :{\text{PU}} \to {\text{A}}$	0.201***	0.014	14.864	< 0.001	Yes
${\text{H}}_{3} :{\text{PEoU}} \to {\text{A}}$	0.772***	0.015	49.841	< 0.001	Yes
${\text{ H}}_{4} :{\text{A}} \to {\text{Intenion}}$	0.657***	0.015	43.129	< 0.001	Yes
${\text{H}}_{5} :{\text{GP}} \to {\text{Intention}}$	− 0.019	0.012	− 1.500	> 0.05	Neutral
${\text{H}}_{6} :{\text{PS}} \to {\text{Intention}}$	0.274***	0.013	20.747	< 0.001	Yes
${\text{H}}_{7} :{\text{Cost}} \to {\text{Intention}}$	0.036*	0.011	3.236	< 0.05	Yes
${\text{H}}_{8} :{\text{SI}} \to {\text{Intention}}$	− 0.027*	0.013	− 2.102	< 0.05	No
${\text{H}}_{9} :{\text{Intention}} \to {\text{AU}}$	0.410***	0.014	27.553	< 0.001	Yes

***p value < 0.001; *p value < 0.05

### Hypothetical test

Because the goodness-of-fit statistics are satisfactory, the overall fit of the model is acceptable. The test results show that among all the nine groups of relationships, six groups are supported, two groups are negative, and one of the two negative groups is neutral, i.e., the results are not significant. The results generally support the proposed model, illustrating the new roles of perceived security and the cost of use in e-cash application.

The specific test results are as follows: Perceived e-cash ease of use has a significant positive effect on perceived e-cash usefulness, as indicated by the coefficient (0.797). The relationship between perceived e-cash usefulness and attitude is supported by a critical ratio (C.R.). The C.R. is a *t*-value obtained by dividing the estimate of the covariance by its standard error (Shin 2009). According to Arbuckle (2005), C.R. values larger than 1.96 and 2.32 are statistically significant at 0.05 and 0.01, respectively. Therefore, perceived e-cash ease of use may result in a higher level of positive attitude toward e-cash. Attitude toward e-cash is a particularly important determinant of user intention for e-cash services.

It is interesting that the impact of perceived government policy is not significant for e-cash acceptance. However, in the context of e-cash usage, it is in line with cognition. In an emerging market, customers pay less attention to government policy about high technology than developed market. These policies are thought to be hard to understand, especially for those with a relatively low level of education.

The perceived e-cash security has a significant positive effect on e-cash use intention. The specified relationship between e-cash use cost and e-cash use intention is supported by the data. However, it is worth noting that the effect of social impacts is negative. Nevertheless, in the context of e-cash, it is understandable. People tend to be relatively cautious about using a new payment method. This is a trust problem. In the process of financial reform, when new business forms appeared, there was always chaos, such as the P2P crisis and the insurance marketing mess. There is always a common denominator in these situations: relationship marketing. The consequences of acquaintance marketing are not ideal, resulting in people's rejection of a new financial production. Therefore, the social impact has a negative impact on consumers’ acceptance of e-cash.

### Analysis of the influence of demographic characteristics

This section uses sample segmentation (Ha et al. 2007; Serenko et al. 2006) to examine the impact of demographic characteristics on consumers' use of e-cash. These characteristics are gender, age, income, and education.

In terms of gender (Table 7), perceived e-cash ease of use has a significant positive effect on perceived e-cash usefulness and attitudes toward e-cash for both men and women. E-cash use intention for the two genders has a positive effect on the actual use of e-cash. Moreover, there is a significant positive effect of attitudes toward e-cash and perceived security on the intention to use e-cash for both men and women. While the e-cash use cost and government policy has no significant effect on male use intentions, they both have a significant negative impact on women. Meanwhile, the social impact has no significant effect on the intent of women, but it has a negative impact on men.

**Table 7:** The impact of gender on e-cash us

Hypothesis	Male			Female		
	Coefficient	C.R	***p*** value	Coefficient	C.R	***p*** value
${\text{H}}_{1} :{\text{PEoU}} \to {\text{PU}}$	0.783	42.061	< 0.001	0.810	41.560	< 0.001
${\text{H}}_{2} :{\text{PU}} \to {\text{A}}$	0.197	10.718	< 0.001	0.208	10.356	< 0.001
${\text{H}}_{3} :{\text{PEoU}} \to {\text{A}}$	0.774	36.925	< 0.001	0.768	33.445	< 0.001
${\text{ H}}_{4} :{\text{A}} \to {\text{Intenion}}$	0.616	28.091	< 0.001	0.702	33.234	< 0.001
${\text{H}}_{5} :{\text{GP}} \to {\text{Intention}}$	0.017	0.891	> 0.05	− 0.035	− 2.164	< 0.05
${\text{H}}_{6} :{\text{PS}} \to {\text{Intention}}$	0.297	15.312	< 0.001	0.239	13.435	< 0.001
${\text{H}}_{7} :{\text{Cost}} \to {\text{Intention}}$	0.069	4.133	< 0.001	− 0.004	− 0.251	> 0.05
${\text{H}}_{8} :{\text{SI}} \to {\text{Intention}}$	− 0.069	− 3.437	< 0.001	0.002	0.101	> 0.05
${\text{H}}_{9} :{\text{Intention}} \to {\text{AU}}$	0.381	19.079	< 0.001	0.444	19.434	< 0.001

As shown in Table 8, most of the statistical results for adolescents are insignificant in terms of age because of the small number in the group, However, like all other age groups, perceived e-cash ease of use had a significant positive effect on perceived e-cash usefulness in adolescents.

**Table 8:** The impact of age on e-cash use

Hypothesis	Age = 1	Age = 2	Age = 3	Age = 4	Age = 5
${\text{H}}_{1} :{\text{PEoU}} \to {\text{PU}}$	0.844***	0.806***	0.714***	0.810***	0.860***
${\text{H}}_{2} :{\text{PU}} \to {\text{A}}$	0.152	0.155***	− 0.011	0.238***	0.217***
${\text{H}}_{3} :{\text{PEoU}} \to {\text{A}}$	0.774***	− 0.021	0.021	0.770***	0.748***
${\text{ H}}_{4} :{\text{A}} \to {\text{Intenion}}$	0.525***	− 0.007	− 0.30	0.637***	0.698***
${\text{H}}_{5} :{\text{GP}} \to {\text{Intention}}$	0.100	0.221***	0.365***	0.016	0.022
${\text{H}}_{6} :{\text{PS}} \to {\text{Intention}}$	0.138	0.332***	0.407***	0.339***	0.146***
${\text{H}}_{7} :{\text{Cost}} \to {\text{Intention}}$	0.028	0.564***	0.403***	0.004	− 0.061
${\text{H}}_{8} :{\text{SI}} \to {\text{Intention}}$	0.020	0.019	− 0.117***	− 0.044	0.113
${\text{H}}_{9} :{\text{Intention}} \to {\text{AU}}$	0.127	0.470***	0.337***	0.338***	0.467***

***p value < 0.001

However, the perceived e-cash ease of use and the perceived e-cash usefulness have significantly positive effects on the e-cash attitudes in groups of older people. The perceived e-cash security is a particularly important determinant of e-cash use intention. E-cash use intention has a significant positive effect on the actual use of e-cash. Other potential variables differ significantly depending on the age group.

In terms of income (Table 9), the perceived e-cash-use has a significant positive effect on perceived e-cash usefulness for all income groups. E-cash use cost has a negative effect on the lower income group (*Income* = 1) and higher income groups (*Income* = 4 and *Income* = 5), while it has a positive effect on the groups with medium income. Government policy also has a negative effect on the lower income group.

**Table 9:** The impact of income on e-cash use

Hypothesis	Income = 1	Income = 2	Income = 3	Income = 4	Income = 5
${\text{H}}_{1} :{\text{PEoU}} \to {\text{PU}}$	0.851***	0.813***	0.796***	0.751***	0.683***
${\text{H}}_{2} :{\text{SI}} \to {\text{Intention}}$	0.193***	0.056	0.106	0.125*	0.114*
${\text{H}}_{3} :{\text{Cost}} \to {\text{Intention}}$	− 0.027	0.013	0.020	− 0.030	− 0.059*
${\text{H}}_{4} :{\text{GP}} \to {\text{Intention}}$	− 0.084	− 0.132***	0.050	− 0.018	0.162***
${\text{H}}_{5} :{\text{PU}} \to {\text{Intention}}$	0.382***	0.429***	0.377***	0.476***	0.180*
${\text{H}}_{6} :{\text{PS}} \to {\text{Intention}}$	0.518***	0.388***	0.262***	0.260***	0.231***
${\text{H}}_{7} :{\text{PEoU}} \to {\text{Intention}}$	0.326***	0.381***	0.449***	0.364***	0.641***

***p value < 0.001; *p value < 0.05

As far as education is concerned, most of the statistical results are not significant for the level of education due to the limited sample size of the bachelor’s degree and above groups as shown in Table 10. For other groups, the perceived e-cash usefulness has a significant positive effect on e-cash use. Perceived e-cash security has a significant positive effect on e-cash use intention while e-cash use intention has a significant positive effect on the actual use of e-cash.

**Table 10:** The impact of education on e-cash use

Hypothesis	Edu = 1		Edu = 2		Edu = 3	
	Coefficient	*p* value	Coefficient	*p* value	Coefficient	*p* value
${\text{H}}_{1} :{\text{PEoU}} \to {\text{PU}}$	0.869	< 0.001	0.760	< 0.001	.780	< 0.001
${\text{H}}_{2} :{\text{PU}} \to {\text{A}}$	0.164	< 0.001	0.207	< 0.001	.380	< 0.001
${\text{H}}_{3} :{\text{PeoU}} \to {\text{A}}$	0.814	> 0.05	0.754	< 0.001	.536	< 0.001
${\text{H}}_{4} :{\text{A}} \to {\text{Intenion}}$	0.533	> 0.05	0.712	< 0.001	.911	> 0.001
${\text{H}}_{5} :{\text{GP}} \to {\text{Intention}}$	0.006	> 0.05	− 0.033	> 0.05	.031	> 0.05
${\text{H}}_{6} :{\text{PS}} \to {\text{Intention}}$	0.286	< 0.001	0.275	< 0.001	.028	> 0.05
${\text{H}}_{7} :{\text{Cost}} \to {\text{Intention}}$	0.024	> 0.05	0.059	< 0.001	− .097	> 0.05
${\text{H}}_{8} :{\text{SI}} \to {\text{Intention}}$	0.071	< 0.05	− 0.078	< 0.001	.084	> 0.05
${\text{H}}_{9} :{\text{Intention}} \to {\text{AU}}$	0.391	< 0.001	0.418	< 0.001	0.217	> 0.05

## Conclusions

Nowadays, the innovation of electronic payment methods reduces the number of spaces where cash is used. Central banks in many countries, such as China, are progressing toward the issuance of digital currency. This could be a sign of society gradually moving toward a cashless society in the future. To achieve this goal, customer acceptance of electronic payments is necessary. E-cash is a kind of digital money. Therefore, the findings of this paper can be useful to e-cash as well as the Central Bank Currency to improve individual acceptance.

### Theoretical implications

The purpose of this study is to propose a comprehensive model to explain individual intention behavior toward e-cash. To meet this goal, perceived security, cost of use, and perceived government policy are incorporated into the UTAUT model. As a result, the predictive model is supported by the SEM test and empirical results, indicating good predictive power toward the behavior intention of e-cash.

This paper contributes to the academic literature on e-cash acceptance by indicating that perceived security and cost of use are essential extensions to the traditional UTAUT model. It means that the perceptions of e-cash security and the cost of using e-cash are key factors for market breakthrough. The current study also finds that the impact of perceived government policy is not significant in e-cash acceptance. Sometimes, policy related to new technology is difficult for people to comprehend, and, therefore, only a few customers pay attention to it. Moreover, it is worth noting that the social impact is negative. Although many research studies deal with the social impact of the adoption of e-payments, few have focused on the chaotic situations that emerge when new business forms appear. Furthermore, consistent with the results in prior studies, the current study confirms that PEOU and PU have a significant positive effect on the intention to use e-cash. These two factors directly affect individual attitudes toward e-cash and, therefore, the intention to use it. Overall, the results shed light on the need to improve e-cash service from the customer’s perspective.

### Practical implications

This study’s findings suggest several types of e-cash promotions. First, this study recommends that researchers and relevant stakeholders not only focus on the technical aspects of e-cash, but they should also attach importance to inadequate user acceptance in e-cash promotion. Understanding user behavior is an efficient way to analyze new technology adoption and to develop an appropriate strategy for optimizing user experiences. In terms of perceived e-cash security, it is of great importance to ensure that perceived security risk and risk control it are aligned with reality. The developers could provide customer guides to help develop the skills and knowledge about the secure use of e-cash in their daily lives.

The finding that perceived government policy does not influence e-cash acceptance indicates that the government may need to introduce corresponding policy interpretations when formulating relevant policies. This would help people get a better understanding of the policy. In addition, gradually decreasing the cost of using e-cash is also helpful. It could be covered in the long run by the reduction of fraud cost from e-cash adoption. Meanwhile, to enhance users’ PEOU and usefulness, more vendors could be encouraged to install e-cash payment terminals and to help customers engage easily in using e-cash.

### Limitations and future studies

The limitations of this study may present opportunities for further research. First, due to the restrictions of data collection, this study was conducted among the Chinese population only. Therefore, it would be helpful to collect data in other countries in future research, and cross-cultural comparisons could also be performed. Second, because the population of e-cash users is currently over several million, the sample size of 4.428 may be disproportionate to the whole population and may lead to a limited generalizability of the findings. Researchers could increase the sample size in the future. Finally, the data of this study were collected before the COVID-19 pandemic. Obviously, the outbreak has had a huge impact on customer lifestyles, including payment methods. Therefore, researchers could explore factors within this specific context and test them. The customers’ acceptance of e-cash, as well as DCEP, might improve under this circumstance.

## Data Availability

The data sets analyzed during the current study are not publicly available but are available from the corresponding author on reasonable request.

## Abbreviations

E-cash: Electronic cash
UTAUT: The unified theory of acceptance and use of technology
SEM: Structural equation model
DCEP: Digital Currency Electronic Payment
TRA: Theory of Reasoned Action
TAM: Technology Acceptance Model
PEoU: Perceived ease of use
PU: Perceived usefulness
A: Attitude to e-cash
SI: Social impact
Cost: Cost of use
PE: Perceived security
GP: Perceived government policy
I: E-cash usage intentions
AU: Actual use behavior

## Footnotes

1 Integrated circuit (IC) cards is the bank card with a “chip”. Considering safety and other factors, Europay, MasterCard, and VISA jointly developed the financial payment standard “EMV”, and has gradually upgraded bank cards from magnetic stripe cards to IC cards, which is called the "EMV" migration, also known as "chip migration." This set of standards innovatively puts chip technology on the bank card, equipping the card with a thick and large-capacity chip, which is safer for payment and helps the bank card performs more functions. China has implemented a chip migration plan since 2011.
